# Using the Radial Shear Rolling Method for Fast and Deep Processing Technology of a Steel Ingot Cast Structure

**DOI:** 10.3390/ma16247547

**Published:** 2023-12-07

**Authors:** Alexandr Arbuz, Anna Kawalek, Alexandr Panichkin, Kirill Ozhmegov, Fedor Popov, Nikita Lutchenko

**Affiliations:** 1 Core Facilities—Office the Provost, AEO Nazarbayev University, 53 Kabanbay Batyr Avenue, Astana 010000, Kazakhstan; nikita.lutchenko@nu.edu.kz; 2 Department of Production Management, Faculty of Engineering Production and Materials Technology, Częstochowa University of Technology, ul. J.H. Dąbrowskiego 69, 42-201 Częstochowa, Poland; kawalek.anna@wip.pcz.pl (A.K.); kvozhmegov@wp.pl (K.O.); 3 Institute of Metallurgy and Ore Benefication JSC, Satbayev University, 29/133 Shevchenko Street, Almaty 050010, Kazakhstan; a.panichkin@satbayev.university; 4 Department of Metallurgy, Faculty of Metallurgy and Mechanical Engineering, Karaganda Industrial University, 30 Republic Avenue, Temirtau 101400, Kazakhstan; fedor_popoff@mail.ru

**Keywords:** rheology, plastometry, radial-shear rolling, severe plastic deformation, FEM-simulation, ingots, casting structure, fine-grained structure

## Abstract

In advancing special materials, seamless integration into existing production chains is paramount. Beyond creating improved alloy compositions, precision in processing methods is crucial to preserve desired properties without drawbacks. The synergy between alloy formulation and processing techniques is pivotal for maximizing the benefits of innovative materials. By focusing on advanced deep processing technology for small ingots of modified 12% Cr stainless steel, this paper delves into the transformation of cast ingot steel structures using radial shear rolling (RSR) processing. Through a series of nine passes, rolling ingots from a 32 mm to a 13 mm diameter with a total elongation factor of 6.02, a notable shift occurred. This single-operation process effectuated a substantial change in sample structure, transitioning from a coarse-grained cast structure (0.5–1.5 mm) to an equiaxed fine-grained structure with peripheral grain sizes of 1–4 μm and an elongated rolling texture in the axial part of the bar. The complete transformation of the initial cast dendritic structure validates the implementation of the RSR method for the deep processing of ingots.

## 1. Introduction

In recent years, there has been a renaissance in the exploration of liquid-metal-cooled fast reactor (LMFR) fuels and fuel cycle possibilities [1]. This renewed interest has not only been observed in various national research and development initiatives, but has also extended to international collaborative efforts. Some of these global undertakings include the International Project on Innovative Reactors and Fuel Cycles (INPRO), the Generation IV International Forum (GIF), and the Global Nuclear Energy Partnership (GNEP) [2,3,4].

Austenitic nickel-based stainless steels in the 300s family were initially chosen because of their good long-term mechanical properties at high temperatures and resistance to sodium coolant [5,6]. However, nickel, which stabilizes the austenite phase and gives steel its heat resistance, so necessary in high-temperature Gen-IV, experiences problems with induced radioactivity [7,8]. Nickel transition under neutron irradiation from the long-lived radioactive isotope nickel-63 makes reactor core structure disposal very problematic [9]. It is also known that matrix precipitates γ′, which gives nickel steels increased strength, are unstable under irradiation [10,11]. This increases brittleness and reduces the strength of grain boundaries of such steel, which were unacceptably high, leading to the development of alternative stainless steels [12,13,14].

Nickel can be replaced by particulate, fine dispersed inclusions such as oxides or carbides. As reinforcing and stabilizing elements, yttrium oxide (Y_2_O_3_) particles are most effective [15,16]. This is the main dispersion strengthened (ODS) steel idea. Moreover, yttrium oxide has increased resistance to radiation [17,18,19,20], thereby prolonging the maximum service life of structural materials, and solving labor-consumption issues and the dangerous processing and disposal of steel structures in decommissioned nuclear power plant (NPP) cores.

ODS steels are usually produced using powder metallurgy by various types of mechanically alloyed powder sintering [21,22]. These methods are associated with problems related to residual porosity, uneven composition, and large grain size of 80 µm or more [23,24]. In addition, billets, as well as ingots, need additional deformational and heat treatments to improve structures.

The production of such special materials, as a rule, is carried out in small-scale production, special enterprises [25,26]. The technological chain usually includes smelting an ingot using a vacuum of at least 10–3 mbar, hot deformation processing (forging, pressing, and rolling), and cold deformation processing (pilger rolling, drawing, etc.) [27,28,29,30]. Due to the high quality requirements of semi-finished and finished products, these technological schemes are characterized by significant metal losses and low yields [31]. As a result, finished products for the nuclear industry will have high costs, thus the development of technologies that reduce these negative consequences is highly relevant.

Even minor changes in chemical composition can affect the manufacturability of a material during product manufacturing, primarily at cold deformation processing stages [32,33]. In addition, as a rule, due to the high costs of smelting full-scale ingots as in mass production, ingots of smaller diameter are used here [34,35]. A decrease in diameter leads to a decrease in the deformation of the cast structure during hot processing, which can lead to structural inhomogeneities during subsequent processing operations [36]. Therefore, deformation processing studies of small ingots of special materials are of great relevance. This is the main focus of this work.

**Figure 1:**
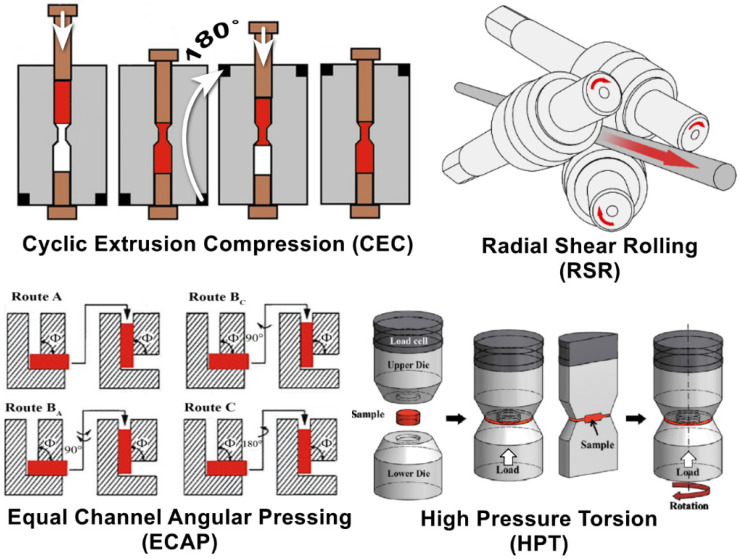
Scheme showing various SPD processes.

For the deep processing of a cast structure, severe plastic deformation (SPD) methods can be used [37,38]. For this initial purpose, the equal-channel angular pressing (ECAP) method was invented by Segal [39,40]. This method later became generally accepted and most widespread in the bulk ultrafine-grained (UFG) and nanostructured material field by SPD [41,42,43]. Various SPD process schemes are shown in Figure 1.

Generally, using plastic strain intensification to improve the quality of ingots makes a lot of sense and has a previous application history. The most common use of SPD methods is in the forging of ingots using special dies [44,45] or in all-round forging [46]. High-effective microstructure processing methods such as high-pressure torsion (HPT) [47,48,49] are unfortunately not applicable outside laboratories, and even more so for ingots, not applicable due to sample size limitations and method scaling factors. The same can be said for cyclic extrusion (CEC) [50,51], accumulated roll bonding (ARB) [52], and some other methods [53,54,55,56,57,58,59]. Of course, ECAP can be used for pressing ingots, but this is not technologically easy due to the need for several pressing cycles and considerable effort and equipment strength requirements. This general point can be seen clearly from the SPD process schemes in Figure 1.

One of the alternative and still not widely used methods of intensive structure refinement using the SPD method is Radial Shear Rolling (RSR) [60,61]. This method is outwardly similar to the classical Mannesmann method for producing seamless pipes [62,63] but differs significantly from it. Figure 1 shows the process scheme.

As can be seen from Figure 1, there are not two skew rolls, like in the pipe piercing scheme, but three skew rolls, and not a pipe is rolled, but a solid bar. A more important difference is not visible in the figure; due to the special combination of roll angles, all-around compressive stresses are realized in the deformation zone, without tensile stresses, as it works in pipe piercing processes. Due to increased roll skew angles of 18–21 degrees, metal flows in the deformation zone have an axisymmetric vortex character with a gradient along the radius [64]. Such a stress–strain state ensures intense deformation of the outer sample zone with the formation of an equiaxed ultrafine-grained structure [65]. Laminar metal flow along the rolling axis in the central third zone of the bar ensures the formation of a rolling texture [66].

This method has been successfully used for the significant refinement of titanium [67,68], zirconium [64,69], copper [70], and steel [71]. In many of these cases, a gradient structure is formed. All experiments with radial shear rolling involve the processing of semi-products of rolling production with a structure either after hot rolling or after recrystallization annealing.

One of the main requirements is to provide a structure. NPP core zone structural elements should be predominantly recrystallized with a grain size of at least 9–12 points according to ASTM E112 [72,73]. Such a structure type can be achieved by the described method, therefore, the use of RSR is justified.

Equally, the influence of radial shear rolling on the structure of ingots is of no less scientific interest. Implemented high levels of accumulated deformation and a stress state scheme, which are favorable for materials with reduced plasticity, promise significant effects with relatively small changes in workpiece dimensions.

The main purpose of this study was to investigate ingot initial structure behaviors under the influence of radial shear rolling. The possibilities of this method were realized by evaluating the processing microstructures of special alloy ingots in small-scale production conditions of NPP critical parts.

## 2. Materials and Methods

**Figure 2:**
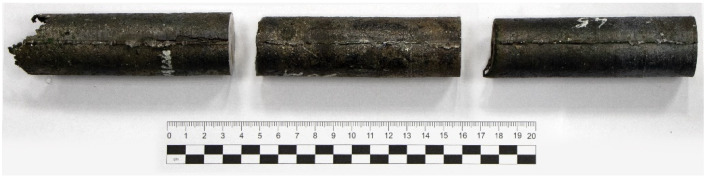
Initial 32 mm diameter ingots.

Yttrium-modified stainless steel small ingots were used as experimental materials for this study. Ingots were obtained by vacuum induction furnace melting as part of other research focused on generating oxide dispersion-strengthened (ODS) steel. The base material for melting was ordinary Fe-13%Cr steel (AISI 403). During the melting process, a small quantity of metallic yttrium and iron oxide (0.5% together) was added to steel. Metallic yttrium undergoes oxidation through the reduction of iron oxide, transforming it into yttrium oxide (Y_2_O_3_) particles in steel. The melting operation was carried out at a temperature of 1600 °C and a furnace pressure ranging from 1 to 5∙10^2^ Pa. The final composition percentages were Fe-86.50, Cr-12.47, Si-0.25, Y-0.12, and Other-0.65. The melting technology and its features, including oxide dispersion and composition fluctuations are other ongoing research subjects and will be published separately. Here, we studied casted structure deep processing. A typical casting dendrite ingot structure was reached for a 32 mm diameter ingot intended for RSR rolling experiments (Figure 2).

**Figure 3:**
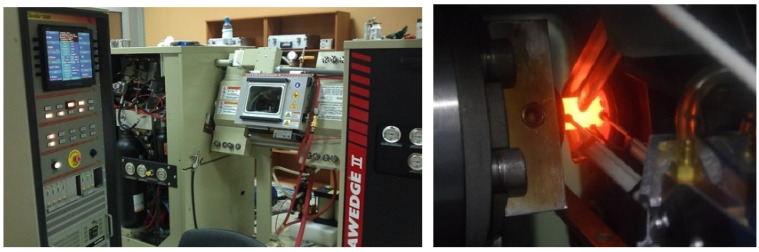
Gleeble 3800 testing machine (**left**) and a photo showing the testing of cast ODS-steel specimens in ISO-T model dies with mounted extensometers for longitudinal and transverse measurements of specimens during testing (**right**).

Casted ingots were examined for rheology properties using a Gleeble 3800 (Dynamic systems Inc., Austin, DX, USA) plastometer using the “Pocket Jaw” module. Plastometric tests were carried out by the uniaxial compression of cylindrical specimens at strain rates of 0.5 s^−1^, 5 s^−1^, and 15 s^−1^ at temperatures of 600 °C, 800 °C, 1000 °C, and 1200 °C. That is, 12 deformation cases of modified steel were studied. Strain rate and temperature ranges were selected based on all-round forging and radial shear rolling conditions of the resulting alloy, and accounted for the thermal effects of plastic deformation. Although the average strain rate during radial shear rolling corresponded to 5 s^−1^, there were regions of localized metal flow where the strain rate was lower (central part of the bar) and higher (periphery). The testing process is shown in Figure 3.

Cylindrical specimens with a working part diameter of 10 mm and a length of 12 mm from several initial ingots were made. The test temperature was controlled using a chromel–copel thermocouple welded to the central part of the specimen on the Therwocouple welder tool supplied with the Gleeble 3800 set. Graphite-based thin gaskets were used as a lubricant in tests. Working dies of the ISO-T model were additionally lubricated with OKS255 (OKS, Maisach, Germany) grease after each test.

Radial-Shear Rolling for ingot processing computer simulations by the Finite Element Method (FEM) using DEFORM-3D (SFTC, Columbus, OH, USA) software were conducted.

A verification experiment was conducted using the RSR-10/30 radial shear rolling mill at Karaganda Industrial University. The rolling process included 10 passes with a 2 mm step (in terms of diameter), commencing from an initial diameter of 32 mm and concluding at a diameter of 13 mm, with an initial heating temperature of 1200 °C. These represented the maximum and minimum achievable rolling diameters for the mill to attain the highest deformation level. Rolling diameters for each pass were the next route (mm): 32-30-28-26-24-22-20-18-16-13. Rolling reduction factor and temperature selections were guided by reference materials and previous studies [74,75,76,77]. The heating process was conducted using the Nabertherm LH-30/14 heating furnace.

**Figure 4:**
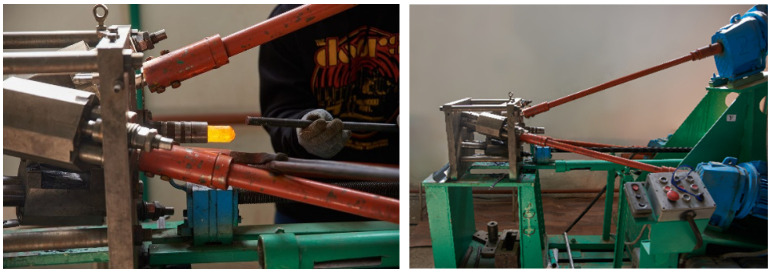
Experimental rolling and the RSR-10/30 radial shear rolling mill.

The experimental rolling process is shown in Figure 4. The RSR mill the next rolling gape diameter setting process to the next pass took 2–5 min. The rolling process is shown in Figure 4.

**Figure 5:**
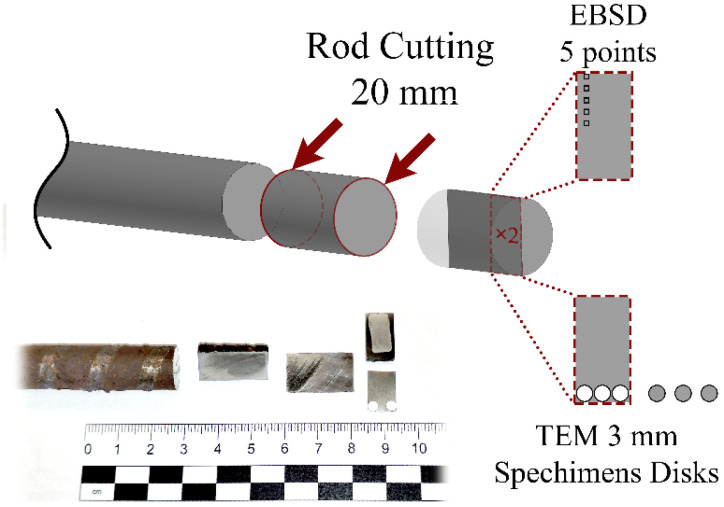
The rolled bar cutting scheme.

After rolling, the bar was cut for microstructure characterization into short small bars, which were cut in half along the axis using a Brilliant-220 (QATM, Mammelzen, Germany) precision cutting machine with a cutting speed of 10 μm/s and intensive water cooling to minimize deformation–temperature damage to structures. From both halves of the section, two thin plates were cut—one for Transmission Electron Microscopy (Jeol, Tokyo, Japan) specimen preparation to characterize deformed peripheral zone fine structure zones. Another plate was used for Electron Backscatter Diffraction (EBSD) sample preparation by electrolytic polishing (jet-polishing). The longitudinal section was preliminarily polished on a Saphire-520 (QATM, Mammelzen, Germany) grinding and polishing machine, and then electrolytically polished on a LectroPol-5 (Struers, Copenhagen, Denmark) unit. A 3 mm diameter round TEM specimen was punched out with a Gatan puncher and subjected to electrolytic thinning on a TenuPol-5 (Struers, Copenhagen, Denmark) machine. In both cases, A2 electrolytes recommended by the manufacturer were used. Electrolytes forced cooling to −20 °C using attached Julabo 600F cryostats (Julabo, Seelbach, Germany). The cutting scheme is shown in Figure 5.

**Figure 6:**
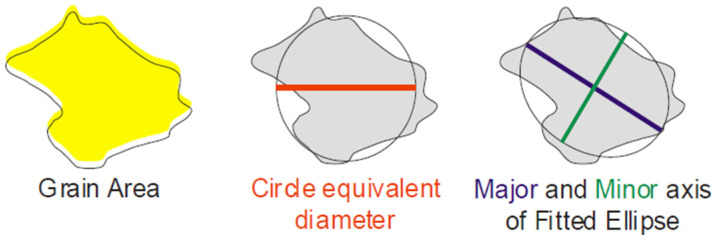
Scheme for calculating grain size parameters.

Electron microscopy methods were used to study microstructural changes. The microstructural characterization of grains, including crystallographic orientation, was performed using Electron backscatter diffraction (EBSD) on a Carl Zeiss Crossbeam-540 field emission scanning electron microscope (Carl Zeiss, Jena, Germany) with an NordlysNano EBSD(Oxford Instruments, Abingdon, UK) attachment. Diffraction pattern recognition and mapping were performed using HKL Channel-5 Tango software v.5.12.74.0 (Oxford Instruments, Abingdon, UK). Maps were built along the radius line from the center to the periphery at five points and at the same distance with steps of 1.625 mm. Once the grains were reconstructed, a variety of grain size parameters were automatically calculated, e.g., Circle Equivalent Diameter (CED) was better and more accurate in characterizing deformed grain sizes. Therefore, we used this parameter later for grain size. Major and minor axis ratios were used to characterize grain elongation parameters. The scheme according to the Channel-5 User Manual is shown in Figure 6.

The fine structures of the most deformed peripheral zones were studied on a JEOL JEM-1400PLUS transmission electron microscope with a Gatan OneView camera (Gatan Inc., Pleasanton, CA, USA) in bright field mode. The original ingot sample microstructure, due to its very large grain size and beyond the minimum field of view, was performed only on a SEM JEOL JSM IT-200LA (Jeol, Tokyo, Japan) in back-scattered electron (BSE) mode.

A microhardness test was performed on the EBSD-ready-cross-section plate using the Shimadzu HMV-G31ST (Shimadzu, Kyoto, Japan) tester and the Vickers method (10 HV) using five times averaging with a load of (2.942) N and 10 s holding times.

## 3. Results and Discussion

### 3.1. Rheological Properties and Database Making

**Figure 7:**
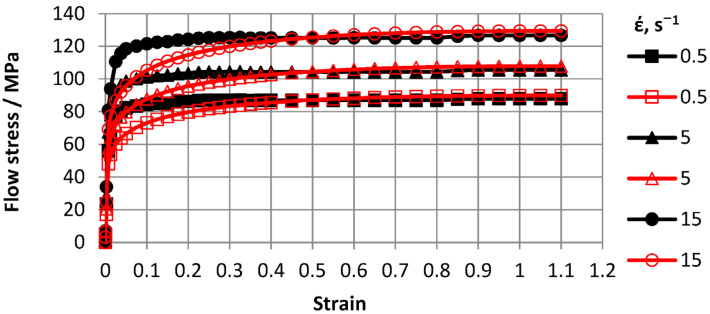
Material flow curves obtained at 600 °C: solid markers—indicate experimental data and transparent markers—–indicate approximation results.

**Figure 8:**
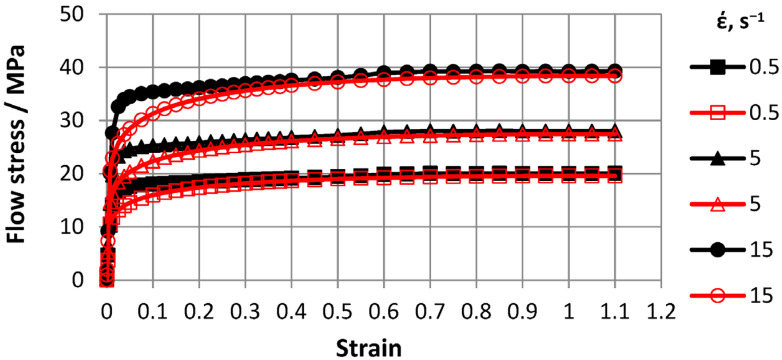
Material flow curves obtained at 800 °C: solid markers—indicate experimental data and transparent markers—indicate approximation results.

**Figure 9:**
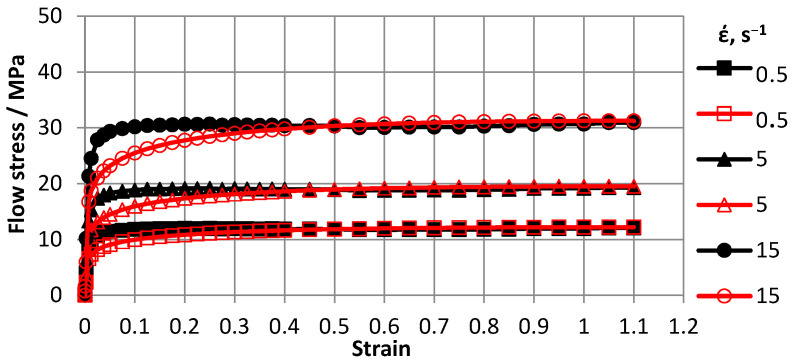
Material flow curves obtained at 1000 °C: solid markers—indicate experimental data and transparent markers—indicate approximation results.

**Figure 10:**
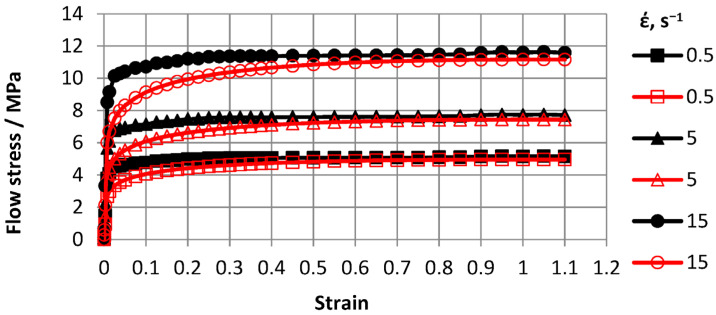
Material flow curves obtained at 1200 °C: solid markers—indicate experimental data and transparent markers—indicate approximation results.

Based on Gleeble 3800 plastometer plastometric test results, ODS steel stress-strain flow curve graphs for 0.5–15 s^−1^ strain rate ranges and 600–1200 °C temperature ranges were plotted. To construct curves, three tests were conducted and a total of 30 tests were carried out. Flow curves are shown in Figure 7, Figure 8, Figure 9 and Figure 10.

The advantage of the compression test scheme was the similarity of the load application scheme with many metal pressure treatment processes and data generation on the resistance of the metal to plastic deformation over a wide range of deformations. The Figure 7, Figure 8, Figure 9 and Figure 10 showed that with increasing temperatures from 600 to 1200 °C, deformation resistance values decreased by more than 10 times. Increasing the strain rate from 0.5 s^−1^ to 15.0 s^−1^ increased strain resistance values, and at T = 600 °C, the increase was approximately 30%. At T = 800 °C, the increase was approximately 48%. At T = 1000 °C, the increase was approximately 57%, and at T = 1200 °C, the increase was 55%. Thus, the effects of increasing strain rates on strain resistance increased with increasing test temperature.

Flow curve characters in temperature ranges coincided. In the strain range up to *ε* = 0.5, flow curves reached steady-state stages. Moreover, with increasing strain rates, eliminated stages occurred at higher strain values.

For the practical use plastometric study results, an approximation of flow curves was carried out using a generalized dependence—the function (1) of Hensel A., Spittel T. [33].

(1) $$\sigma_{p}=Aexp\left(m_{1}T\right)T^{m_{9}}\varepsilon^{m_{2}}exp(m_{4}/\varepsilon ){\left(1+\varepsilon \right)}^{m_{5}T\;}exp(m_{7}\varepsilon ){\dot{\varepsilon }}^{m_{3}}{\dot{\varepsilon }}^{m_{8}T}$$

where *A* and *m*_1_…*m*_9_—are unknown coefficients in the deformation resistance model.

To solve the regression, we used the built-in program of the Forge 2008^®^ “RheologyDatabase” program, which used the Levenberg–Marquardt method. This method provides the automatic determination of coefficients based on an array of experimental points. The operation was performed at the Institute of Metal Forming Processes and Safety Engineering, Czestochowa Polytechnic (Poland). The approximating coefficients were as follows: *A* = 2251; *m*_1_= −0.0052; *m*_2_ = −0.01; *m*_3_ = 0.2; and *m*_4_ = −0.05. An analysis of the verification results of approximated and real results showed an approximation error of no more than 10%. The approximation was considered optimal when the approximation error was not more than 15%. The difference between flow curves obtained experimentally and calculated by deformation *ε* < 0.4 was due to the fact that deformation resistance *σ_p_* values were influenced by elastic deformation, which was not accounted for in Equation (1). Thus, in experimental flow curves, plastic deformation began at large deformation resistance *σ_p_* values. As the degree of deformation increased, the error decreased. Due to the large magnitude of plastic deformation that occurred in simulated processes, the difference between the magnitude of the actual deformation and the value obtained after approximation in the range of smaller deformations of 0.4 did not affect the results.

After processing the data, a new material database was created for a new material base was created for Deform-3D suitable for modeling deformation processes of the cast ODS-steel in the temperature range of 600–1200 °C and strain rates of 0.5–15 s^−1^.

The database can be used to simulate hot deformation processing conditions (hot pressing, hot forging, and hot rolling), which are used at the beginning of the technological cycle for the manufacture of fast reactors fuel assemblies, fuel element claddings, and plugs.

### 3.2. Computer Simulation of RSR Ingot Processing

**Figure 11:**
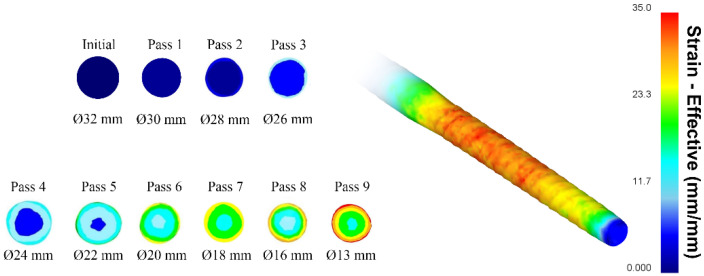
Computer simulation of RSR ingot processing.

Radial shear rolling processing was simulated for the sequential rolling of a 32 mm diameter ingot to a 13 mm diameter bar in nine passes with a 2–3 mm reduction in diameter on the RSR-10/30 (MISIS, Moscow, Russia) rolling mill. The total rolling elongation factor was 6.05. Simulation results are shown in Figure 11.

The accumulated total strain degree in some areas of the final bar reached 30 mm/mm with a minimum value of 15 mm/mm. Such deformation levels should be sufficient for initial casted structure deep transformation. Strain levels had axisymmetric radial gradients regarding bar cross-sections. These differences can be used to interpret microstructural changes.

### 3.3. Microstructural Change Study

**Figure 12:**
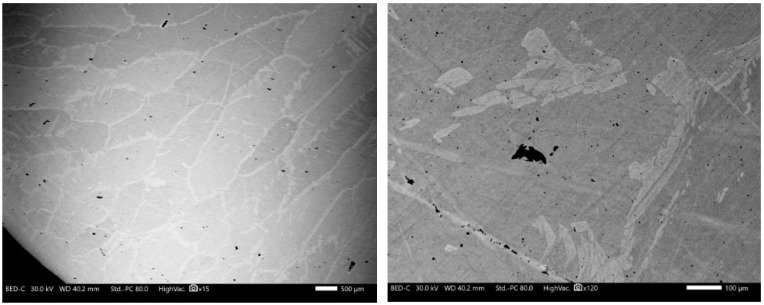
SEM (BSE) scale images of original ingot dendritic structures.

Initial ingot microstructures at various magnifications are shown in Figure 12. The images clearly show the light axes of dendrites and darker interdendritic spaces. The grain sizes fall within the range of 0.5–1.5 mm. Consistently, dendritic cast structures are prevalent throughout the steel volume, embodying typical ingot structures as expected. This microstructure was suitable for casted structure evolution research during RSR processing.

**Figure 13:**
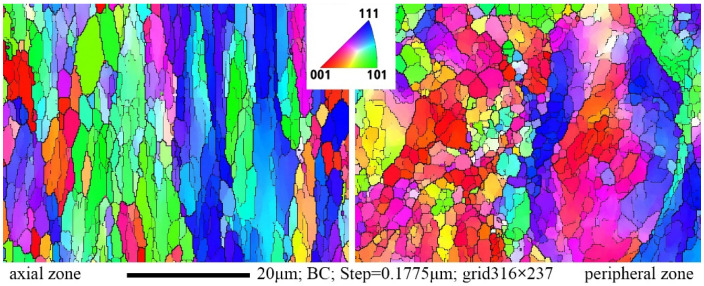
EBSD map of the axial zone (**left**) and peripheral zone (**right**) of a longitude bar section after final rolling.

Following the rolling process, a gradient structure was expected. To characterize this, gradient EBSD mapping was used. The mapping spans along the longitudinal section radius from the axial zone to the peripheral zone at equal distance measurements at five specific points. The resulting EBSD maps, showcasing full-size microstructure images of the most differenced axial and peripheral zones of the bar (left and right images respectively), are presented in Figure 13.

The peripheral zone map of the bar revealed a fine-grained structure characterized by equiaxed grains with diverse orientations. Within structures, similar orientation grains to elongated along rolling direction chains aligned and interspersed randomly, exhibiting significantly different orientation grain chains. The occurrence of identical orientation grain chains suggested unfinished potential dynamic recrystallization processes. However, this fact generally correlated with well-known work on radial shear rolling [24,25,26,27,28,29,30,51,52] and SPD processing generally [4,5,6], and indicated significant processing of the cast structure with its complete transformation into fine-grained structures close to nuclear engineering needs [53,54].

The center of the bar microstructure exhibited predominantly similar orientations of large grains elongated in the rolling direction. However, there were notable regions with fine grain clusters and varying orientation zones, rendering the structure more intricate than anticipated based on existing research. It did not resemble a heavily deformed laminar shape typically observed in metals with a strong rolling texture, as previously seen [18,26]. Instead, it appeared to represent an intermediate state between the texture and structure of the bar’s center after potential deformation using the RSR method, as outlined elsewhere [51].

This complex structure likely resulted from a combination of processes involving dynamic recrystallization, manifesting as clusters or colonies of fine grains within the matrix with different orientations, and static recrystallization, leading to the formation of large grains that retained their longitudinal orientation in the rolling direction. However, these large grains were now irregular and more rounded in shape, exhibiting evident misorientation. This phenomenon was probably induced by longer, smoother deformational heating in the central part, contrasting with rapid heating and cooling cycles experienced by the near-surface layer due to large shear deformations and subsequent swift cooling from cold rolls and the environment.

**Figure 14:**
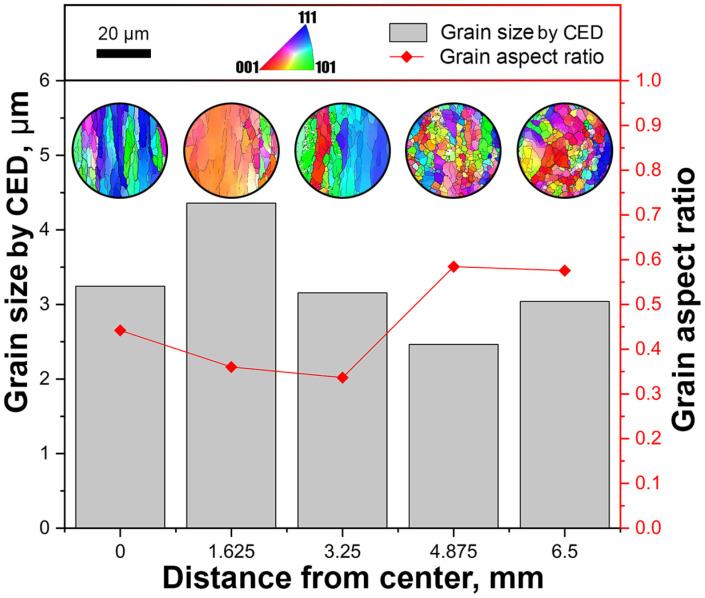
EBSD study of the gradient microstructures of rolled bar longitudinal sections.

To conduct a comprehensive and quantitative analysis of microstructure changes across cross-sections, all EBSD maps were meticulously processed and subjected to statistical analysis. This analysis generated a graph featuring two primary indicators, as depicted in Figure 14. The gray columns, with a scale on the left, represented the average grain size by CED in the axial-peripheral direction. Concurrently, the red plot, with a scale on the right, illustrated the average ratio of the larger and smaller axes of grain dimensions, serving as a measure of equiaxiality. A value close to 1 indicated a more equiaxed grain, while a value closer to 0 suggested a more elongated grain, characteristic of rolling texture morphology. This indicator was introduced to address gradient structures across the rod’s cross-section and to provide a more objective characterization of microstructures than the average grain size by CED alone.

In the axial zone of the rod, the grain ratio value was 0.58, showcasing a 23% change towards the peripheral zone. This indicated a decrease in grain equiaxiality from the peripheral to the axial zone. As illustrated in Figure 13, this aligned with the transition from a fine-grained structure in the peripheral zone to an elongated fine-grained structure along the axis of the bar.

In the provided miniatures, it was evident that grains tended to cluster in fine-grained groups with closely aligned orientations across almost all images. A notable shift in grain equiaxiality was observed around the halfway point of the rod’s radius. The highest concentration of elongated grains started from this region. However, interestingly, the average grain size remained comparable to that of the periphery. Grain growth was initiated at a depth of approximately 2/3 of the radius from the surface. These data were comparable with FEM-simulation data (Figure 10). They had a general gradient similarity regarding the structure-changing radial distribution. A higher total strain level of 25 mm/mm and more for outer zones corresponded to a better structure. Medium 18–24 mm/mm levels corresponded to a transition structure. Although the average grain size at this stage was still notably smaller than the original sample’s grains, which were larger than 1000 µm, the change was considerable. The average grain size across the entire cross-sectional area of the rolled sample was 3.2 μm, representing a reduction of over 300 times compared to initial grain size.

Overall, the cast structure of ingots had a complete transformation throughout the entire cross-section, leading to the successful application of the RSR method for ingot deep processing. The transformed structure of the periphery zone mainly corresponded to ASTM E112 requirements [72,73].

**Figure 15:**
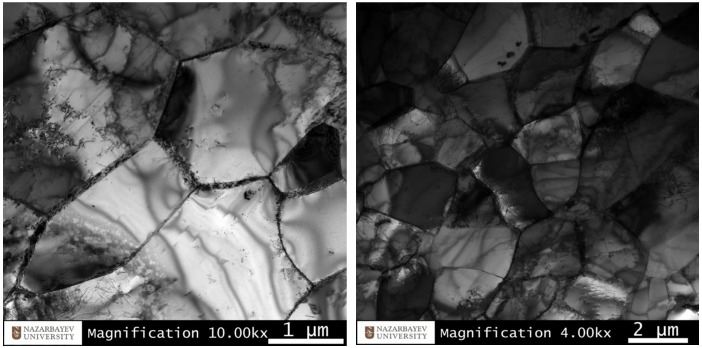
Fine structure of fine-grained zones in the peripheral bar part after final rolling.

The fine structure TEM characterization of the longitudinal section peripheral part (2 mm from the edge along the radius of the rod) at various magnifications is shown in Figure 15. The TEM study affirmed grain refinement down to sizes of 1–4 μm in the peripheral part of the rod, accompanied by a similar misorientation level. Dislocations were primarily concentrated at grain boundaries. Within the grain body, dislocation clusters, if present in sufficient quantities, demonstrated a trend towards dislocation rearrangements, resulting in the formation of a cellular structure. Simultaneously, numerous grains displayed a small number of dislocations, suggesting increased material plasticity. High deformation heating probably triggered fine structure recovery processes with dislocation annihilation. Notably, these observations slightly deviated from previous data [52], which indicated a significantly higher number of dislocations in peripheral ultrafine-grained (UFG) zone grains.

In this case, despite substantial processing, the peripheral region did not transition to the Ultrafine-Grained (UFG) state. Probably, between passes, recovery and recrystallization processes had likely occurred. Specific thermal and rolling conditions played crucial roles in achieving desired microstructural outcomes. Further optimization of the processing parameters may be necessary to achieve a targeted UFG state in the peripheral zone.

Microhardness evaluations showed decreasing values from 212 HV to 183 HV. These changes corresponded to microstructural changes in the full transformation of non-plastic casted structures to normal. Also, a decrease in hardness was explained by dislocation-free grains according to the TEM study.

## 4. Conclusions

Based on our analyses, the following conclusions were formulated:

1. The use of radial shear rolling processing led to changes in the structure of a small ingot of stainless steel modified with yttrium.
2. The original coarse-grained dendritic structure of casted ingots was significantly refined to a fine-grained structure. The region farthest from the axis of the rod, constituting 1/3 of the radius of the rod, had a fine-grained structure with a grain size of 1–4 μm, with an equiaxial structure, without a clear dominant orientation structure. The axial zone, which also occupied 1/3 of the radius of the rod, had a more elongated and coarse-grained structure with grain sizes from 4 to 10 μm. The predominant grain orientation in this region was oriented along the rolling axis. In the middle region, there was a transition-type structure between these two. The average cross-sectional grain size was approximately 3.2 microns, which was 300 times smaller than grain sizes before the rolling process.
3. Grains in the highly deformed peripheral zone had a relatively small number of dislocations. Based on fine structure and some EBSD features, we conclude that processing can be improved.
4. Resulting blanks were close to nuclear engineering needs and could be used as semi-product improvements for the manufacture of some structural elements for nuclear power plant cores.

## Data Availability

Data are contained within the article.
